# Exploring the Association between Diagnostic and Therapeutic Radiation and the Incidence of Vestibular Schwannoma: A Case–Control Study

**DOI:** 10.1055/a-2741-3551

**Published:** 2025-11-25

**Authors:** Idit Tessler, Angela Chetrit, Nir A. Gecel, Gilad Twig, Avital Perry, Amit Wolfovitz

**Affiliations:** 1 Department of Otolaryngology Head and Neck Surgery, Sheba Medical Center, Ramat Gan, Israel; 2 Faculty of Medicine, Tel Aviv University, Tel Aviv, Israel; 3 Cancer & Radiation Epidemiology Unit, Gertner Institute for Epidemiology and Health Policy Research, affiliated with Tel-Aviv University, Chaim Sheba Medical Center, Tel-Hashomer, Ramat Gan, Israel; 4 Department of Neurosurgery, Sheba Medical Center, Ramat Gan, Israel

**Keywords:** vestibular schwannoma, diagnostic radiation, therapeutic radiation, case–control study, ionizing radiation

## Abstract

**Introduction:**

Ionizing radiation is a known risk factor for various neoplasms, yet its link with vestibular schwannoma (VS) remains unclear. Given that VSs are benign tumors of the eighth cranial nerve, elucidating potential associations with radiation is of clinical interest. This study investigated the association between diagnostic and therapeutic head and neck radiation exposure and VS.

**Methods:**

In a case–control design, we enrolled 137 patients with VS, matched by age and sex with 659 controls. Data were obtained through structured interviews, capturing sociodemographic factors and history of therapeutic head and neck radiation, as well as imaging examinations (X-ray, computed tomography [CT], and magnetic resonance [MR], excluding the last 2 years). Weighted distributions were used to account for up to six controls per case. We used conditional logistic regression to estimate odds ratios (ORs) and 95% confidence intervals (CIs).

**Results:**

The mean age of participants was 53 ± 14.6 years, with 50.4% females. An initial significant association was observed between therapeutic radiation and VS (adjusted OR = 4.94, 95% CI: 2.49–7.98). However, excluding participants who recently underwent radiation therapy attenuated this association (adjusted OR = 2.32, 95% CI: 0.59–9.07; *p* = 0.22). No significant associations were found for diagnostic imaging (ORs of 1.04 [0.86–1.25], 1.18 [0.73–1.92], and 1.19 [0.57–2.49] for X-ray, CT, and MR, respectively).

**Conclusion:**

Our findings do not support a significant relationship between either therapeutic or diagnostic head and neck radiation exposure and the risk of VS, once recent treatments are excluded. Additional large-scale studies are necessary to confirm these observations and to examine potential dose–response effects.

## Introduction

Extensive research has explored the epidemiology and etiology of brain tumors, with particular attention given to the role of radiation exposure. 1 2 3 4 Moderate-to-high-dose ionizing radiation has been firmly established as an environmental risk factor for central nervous system (CNS) tumors. 1 3 5 6

Brain tumors encompass a heterogeneous group of neoplasms, each with distinct etiologies, biological behaviors, clinical courses, pathologies, and morphologies. Among the various subtypes of CNS tumors, schwannomas are recognized as one of the three most prevalent types. 2 Vestibular schwannoma (VS), a slow-growing benign tumor of the eighth cranial nerve, is the most common tumor in the posterior cranial fossa, comprising about 90% of the tumors in this area. 7 However, despite the existing literature on radiation exposure and brain tumors, limited emphasis has been placed on investigating the specific relationship between radiation exposure and VSs. Furthermore, previous studies often did not differentiate between different types of radiation treatments.

As a consequence, the precise link between radiation exposure and the risk of developing VSs remains inadequately established. To address this research gap, the current study aims to investigate the association between diagnostic and therapeutic radiation exposure and the risk of VS development. By distinguishing between these two types of radiation exposure, we seek to enhance our knowledge of the specific effects of each on the incidence of VSs.

## Methods

After receiving IRB approval, we conducted a case–control study of patients who were diagnosed with VS between 2001 and 2004 and age- and sex-matched controls.

Data were collected through personal interviews, as part of the INTERPHONE study, 8 details on the methodology were described in previous publications. 9 10 The present study comprised the Israeli cohort included in the INTERPHONE extended with cases diagnosed at age 60 and above with acoustic neuroma. Controls were identified from the National Population Registry and individually matched by sex, age, and ethnic origin. A post hoc matching was used to assign a maximal number of available controls to each case. The interviews covered, among many other topics, sociodemographic characteristics: Gender, age, ethnic origin, marital status, employment, and smoking; as well as lifetime history regarding radiation exposure for both diagnostic and therapeutic purposes to the head and neck, stratified to X-ray examinations, computer tomography (CT) scans, and magnetic resonance (MR). X-ray procedure was asked for the skull, neck, teeth, full mouth and salivary gland, and blood vessels in the head, neck, or brain. CT scans covered scans of the brain, head, or neck. Data on MRI refer to the scan of any part of the body.

To minimize reverse causation bias—whereby early symptoms of VS might lead to increased diagnostic or therapeutic procedures—we excluded records of diagnostic radiation exposures that occurred within the 2 years prior to the diagnosis (or the reference date for controls). This lag period was selected based on the slow-growing nature of VSs and consistent with prior studies examining similar exposure–outcome relationships. 11 12 13

### Statistical Methods

Distributions among controls were weighted by the inverse number of controls in matched sets that varied up to six controls per case. Case–control comparisons were performed by use of the weighted chi-square test for categorical variables and the weighted *t* -test for continuous variables. Odds ratios (ORs) and 95% confidence intervals (CIs) were estimated using conditional logistic regression for matched sets.

## Results

Our cohort consisted of 137 cases and 659 matched controls. The mean age of cases was 53 ± 14.6 years, with a similar sex distribution (50.4% females) between cases and controls. Demographic characteristics of acoustic neuroma cases and controls are presented in Table 1 . Cases reported lower levels of education than controls (29% vs. 38% with academic education, *p* = 0.1) (not shown).

**Table 1:** Demographic characteristics of acoustic neuroma cases and controls

Characteristics	Cases		Controls		*p* -Value
	*n*	Percentage	*n*	Weighted percentage	
Total	137	100	659	100	–
Gender					
Men	68	49.6	322	50.0	–
Women	69	50.4	337	50.0	
Age					
Mean ± SD	53.0 ± 14.6		53.1 ± 6.5		–
Origin					
Asia	13	9.5	59	8.9	0.9
North Africa	19	13.9	89	14.1	
Europe/America	54	39.4	267	40.0	
Israel	51	37.2	244	37.0	
Regular smoker					
Yes	61	44.5	341	51.5	0.2
No	76	55.5	318	48.5	

Distribution of cases and controls by type of exposure to radiological diagnostic examinations and ORs is presented in Table 2 . Excluding exposure to radiological diagnostic examinations performed within 2 years before diagnosis, acoustic neuroma cases reported slightly more X-rays, CT scans, MRI, and thyroid scans than their gender and age-matched controls. We did not find a significant association between diagnostic radiation exposure and the incidence of VS (OR of 1.04 [0.86, 1.25], 1.18 [0.73, 1.92], and 1.19 [0.57, 2.49] for X-ray, CT, and MRI, respectively).

**Table 2:** Exposure to radiological diagnostic examinations among cases and controls (excluding those performed within 2 years before the diagnosis)

	Cases ( *n* = 137)		Controls ( *n* = 659)		OR	95% CI
	*n*	Percentage	*n*	Weighted percentage		
X-ray skull	36	26.8	164	25.5	1.03	0.69–1.53
X-ray neck	25	18.3	145	21.8	0.84	0.52–1.35
X-ray full mouth	70	51.1	308	45.5	1.12	0.88–1.43
X-ray salivary glands	0	–	5	0.7	–	–
X-ray blood vessels	5	3.7	6	1.0	3.78	0.57–25.15
X-ray	85	62.0	399	59.6	1.04	0.86–1.25
CT scan	29	21.2	123	17.9	1.18	0.73–1.92
MRI	14	10.2	58	8.6	1.19	0.57–2.49
Thyroid scan	7	5.1	27	3.9	1.30	0.43–3.90
Any irradiation scan	92	67.2	432	64.9	1.05	0.82–1.34

In total, 18 cases (13.1%) were exposed to therapeutic radiation compared to 20 (weighted % of 2.9) controls, *p* = 0.002. The case's etiologies for radiation therapy included malignancy (50%), tinea capitis (27.8%), other skin conditions (5.5%), and others (16.6%).

We found a positive association between therapeutic radiation exposure and VS, with participants who received head and neck radiation therapy having a higher risk (crude OR and 95% CI: 4.49 [1.57–12.89], gender, age, and education adjusted OR and 95% CI: 4.94 [2.49–7.98]). However, after excluding patients who received radiotherapy within 2 years of diagnosis, the results became insignificant (adjusted OR and 95% CI: 2.32 [0.59–9.07]; *p* = 0.22). After exclusion, the exposure groups for this analysis became relatively small, with only 7 exposed cases and 17 exposed controls. The causes or medical conditions for radiation therapy are presented in Table 3 . This limited sample size results in low statistical power, based on power calculation and effect size found for other tumors in previous literature. Consequently, the non-significant findings after adjustment should be interpreted cautiously.

**Table 3:** Medical conditions leading to radiation therapy a

	Cases ( *n* = 7)	Controls ( *n* = 17)
	*n*	*n*
Tumors and cancers of the head and neck	0	1
Tinea capitis or ringworm of the scalp	5	11
Other skin conditions	1	2
Infections	0	1
Other	1	2

a Excluding patients receiving radiotherapy within 2 years prior to the diagnosis.

## Discussion

The incidence rates of VS have been steadily increasing, with an estimated rate of 3 to 5 cases per 100,000 person-years. 14 15 16 17 18 19 While the increase in incidence can largely be attributed to enhanced detection, studies have aimed at identifying risk factors. 20 At present, no modifiable risk factors for VS have been conclusively identified. Although certain factors, such as cellphone use and noise exposure, have been proposed as potential risk factors for VS, the connection between these factors and VS development remains controversial due to limited evidence and study design biases. 21 22 23 24 Furthermore, multiple studies have identified smoking as a negative (i.e., protective) risk factor. 25 26 27 28 However, the limited benefit of smoking as a preventive measure must be weighed against the substantially higher risk of developing other forms of cancer and severe medical conditions associated with smoking.

Ionizing radiation is a known risk factor for the development of various neoplasms. 1 2 3 4 However, in the current study, we did not identify a significant association between either diagnostic or therapeutic radiation and the incidence of VS. Our findings are consistent with those of the INTERPHONE study group, which also reported no link between head and neck diagnostic radiography and the occurrence of VS. 29

Although prior research has established a connection between head and neck diagnostic X-rays and the development of meningiomas and gliomas, these studies have not demonstrated a similar link for VS. 30 However, conflicting data exist. A study conducted in Brazil observed a substantial risk of developing VS with exposure to a high volume of cranial X-rays (OR 4.55; 95% CI, 1.10–19.2). 6 The topic of dental X-rays has been particularly contentious. Several studies have reported an association between annual dental X-ray exposure and VS, 31 32 while others have refuted this relationship. 33 34 It should be noted that advancements in technology have led to a dramatic reduction in radiation emitted by dental X-ray machines, which could account for these inconsistent outcomes. 35

Studies investigating the relationship between therapeutic radiation to the head and neck and VS are scarce. For other tumors, therapeutic radiation has been associated with an elevated risk of developing meningiomas (OR 3.7, 95% CI 1.5–9.5) and other neoplastic conditions. 36 One particular study reported a relative risk of 18.8 for nerve sheath tumors among patients treated with an average dose of 1.5 Gy for tinea capitis. 37 Consistent with our findings, other studies have also reported no significant link between therapeutic radiation and VS. 33 35 Advances in therapeutic radiation technology have similarly reduced emitted radiation doses, which could explain these variable results. 35

Although our analysis showed a significant association between therapeutic radiation and VS (adjusted OR = 4.94), this relationship diminished and became statistically non-significant after excluding radiation exposures that occurred within 2 years of diagnosis (adjusted OR = 2.32; *p* = 0.22). Consistent with previous studies, 8 35 we made the deliberate choice to exclude cases of radiation exposure occurring within the 2 years prior to the diagnosis of VS. This decision was informed by the slow-growing nature of VSs, which often take several years to manifest clinically. Given this extended latency period, it is unlikely that radiation exposure in the short term, particularly within the 2 years leading up to diagnosis, would be the causative factor in tumor development. By excluding such recent exposures, we aimed to minimize the potential for reverse causality or lead-time bias, where the tumor itself might prompt medical investigations that include radiation-based diagnostic procedures. 11 12 13 While this approach enhances methodological rigor, it also reduces the number of exposed subjects, which may limit statistical power. Therefore, both sets of findings—before and after exclusion—should be interpreted within the context of these methodological considerations.

Several limitations warrant consideration in interpreting the results of this study. In addition to the relatively small cohort size, the case–control design introduces the potential for recall bias, as participants may struggle to accurately remember their past radiation exposures. Additionally, the retrospective nature of our study—using data from patients diagnosed with VS between 2001 and 2004—limits the applicability of our findings to more recent advancements in radiation technology and exposure patterns.

In conclusion, our study suggests no significant relationship exists between diagnostic or therapeutic radiation exposure to the head and neck and an increased risk of developing VS. Further research is required to confirm these findings and explore any dose–response relationship.
